# Development and Formative Evaluation of a Death Education Program for Community-Dwelling Chinese Older Adults

**DOI:** 10.1093/geroni/igab046.072

**Published:** 2021-12-17

**Authors:** Mandong Liu, Iris Chi

**Affiliations:** 1 University of Southern California, Hangzhou, China (People's Republic); 2 University of Southern California, University of Southern California, California, United States

## Abstract

Planning for end-of-life (EOL) care in advance can enhance one’s quality of life at EOL. Culturally sensitive educational programs are needed in Chinese populations to enlighten the public and encourage advance planning due to a culture of death-denying and avoidance. This study describes the team’s efforts to develop and formatively evaluate a death education program designed for community-dwelling Chinese older adults. The program was designed based on the Knowledge-Attitude-Behavior Model, as a 2-session 3-hour program spreading over two days with 1.5 hours for each day. The content paid attention to discussing the importance of making plans for EOL in Chinese culture and discussing how to have death-related conversations with the family and health care professionals. In 2020, semi-structured interviews were conducted by phone with 12 health care professionals and researchers, and four Chinese older adults in China to obtain their feedback on program content and delivery. The directed content analysis method was used to analyze the data. Although they confirmed multiple challenges in conducting death education in China, such as family avoidance even if an older adult initiates the death-related conversation, health care professionals not feeling comfortable with such discussions, etc., they also felt the urgency and importance of delivering death education among older adults and in society as a whole. Detailed suggestions were categorized into relationship building, program preparation (e.g., setting, materials), multiple ways of recruitment, target population, length, various formats of content delivery, content (e.g., pay attention to spiritual care), and general support from the public.

